# Hydrophobia among the Dogs in New York

**Published:** 1881-10

**Authors:** 


Hydrophobia Among the Dogs in New York.—For the first time since the establishment of the dog pound in this city, a case of hydrophobia has been found. It occurred in a half-Spitz dog, which was found on the streets, September 22d. The case was diagnosed at once by Dr. Ennery, veterinary surgeon. As stated by him, the dog showed very marked evidences of the disease. The animal was put in the dog cart, where he crept about slowly on his belly. The moment his nose touched anything, he would give a convulsive snap. He was evidently stone blind, and his eyes were deeply congested. The other dogs, as is usually the case, seemed afraid of him, and tried to keep out of his way, but he bit many of them before reaching the pound. The diseased animal was shot at once, and those bitten were drowned.

There is a prevalent error regarding the frequency of hydrophobia. This is the first case that has been found among forty-seven thousand dogs that have been drowned at the dog pound in the past five years.

